# Fast free-breathing planning in cardiac MR imaging

**DOI:** 10.1186/1532-429X-11-S1-P127

**Published:** 2009-01-28

**Authors:** Tamer A Basha, Monda L Shehata, Robert G Weiss, Nael F Osman

**Affiliations:** Johns Hopkins University, Baltimore, MD USA

**Keywords:** Cardiac Magnetic Resonance, Cardiac Magnetic Resonance Imaging, Cardiac Phasis, Planning Step, Spiral Acquisition

## Introduction

Cardiac magnetic resonance imaging is an excellent tool for assessing global and regional function of the heart. It is now regarded as the gold standard for the analysis of ejection fraction, ventricular volumes and for quantification of myocardial wall-motion [1]. However, the initial planning step is time consuming and requires at least three or four breath-holds in order to get to the first targeted slices.

## Purpose

In this work, we propose a fast black-blood pulse sequence which requires a scan duration as short as one heartbeat.

## Methods

### Pulse sequence

**Figure 1:**
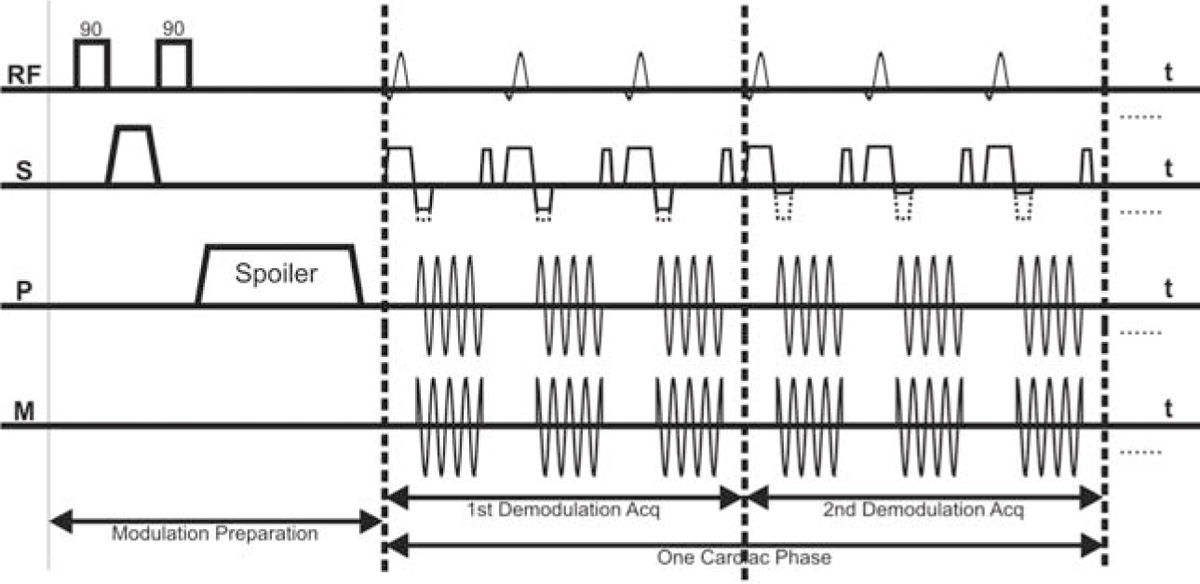
**Pulse sequence diagram.** Dotted lines in the gradient lobes represent the original gradient values without the demodulation

Based on the modified STEAM sequence proposed in [2], Figure 1 shows a diagram for the proposed sequence. First, modulation pulses are applied upon the detection of the QRS complex of the electrocardiogram (ECG). Then images are acquired at a demodulation frequency equal to the original one. This results in obtaining signal only from the tissues that maintained the original modulation and nulling the blood signal because of its flow. A reduced field of view (FOV) is achieved by localizing the modulation pulses such that only the region around the heart is modulated. Then, images are obtained using multi-shot spiral acquisition. The combination of reduced FOV and spiral acquisition allow for acquisition of a complete set of images in just one cardiac cycle.

### In vivo experiments

During regular cardiac imaging exams on a 3 T MRI scanner (Philips Medical Systems, Best, the Netherlands), the proposed sequence was used for free-breathing image planning on fifteen healthy volunteers. All subjects were imaged in the supine position. Following standard survey images using a scout sequence, two series of planning acquisitions are acquired using regular breath-hold cine and the proposed free-breathing technique. Each planning series starts with pseudo 2-chamber then pseudo 4-chamber and ends with the true short-axis plan. A true four-chamber plan was acquired depending on the need of this plan for the running exam.

The following acquisition parameters were used for the proposed technique: FOV, 256 × 256 mm^2^; Excited region width, 200 mm; matrix size, 64 × 64; last flip angle [2], 30°; slice thickness, 10 mm; in-plane spatial resolution, 4–4 mm^2^. For each cardiac phase, two demodulation images were acquired, for each one 3 spirals were used with acquisition window of 7 msec. This results in a temporal resolution of 42 msec. Depending on the heart rate, 17–25 cardiac phases were acquired. The total scan time was 0.7–1.0 sec.

## Results

**Figure 2:**
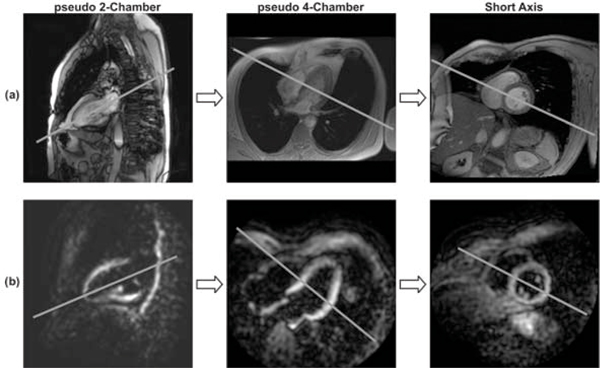
**Planning steps using a) Regular Cine, and b) Proposed technique**.

**Figure 3:**
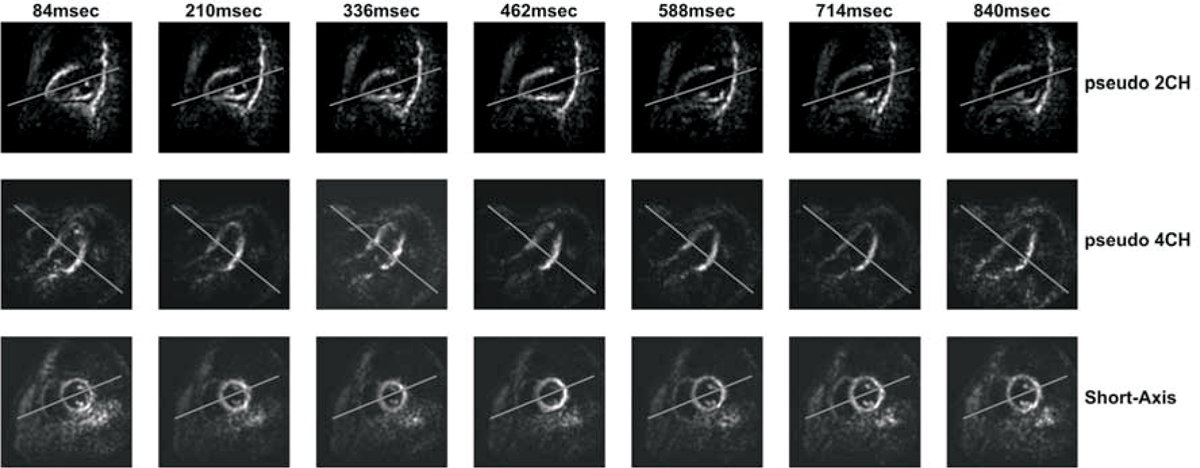
**Representative cardiac phases for planning results using the proposed technique**. Columns represent time frames while rows show different planning slices.

Figure 2 shows representative results for the planning steps for one of the volunteers. While the regular cine images have better resolution and more detail, the images from the proposed technique have enough detail for adequate planning. The black blood feature allows for better recognition of the ventricles. Figure 3 shows the complete cardiac phases of each of these planning steps. The interleaving between the two demodulation images helps in getting clear images over the whole cardiac cycle, either during relaxation (where the first demodulation image has more information) or during contraction (where the second one has clearer ventricle view).

With the same operator, planning time was reduced from 5 ± 2 min using regular cine images for planning to 2.2 ± 0.7 min using the proposed protocol.

## Conclusion

With a relatively low spatial resolution (4 mm), the proposed protocol can be used for cardiac imaging planning without any breath-holds. This helps to reduce the overall scan time and the number of breath-holds in during the exam which is especially important for dyspneic patients.

## References

[1] Yang PC (1998). J Am Coll Cardiol.

[2] Fahmy AS (2006). MRM.

